# CNT Conductive Epoxy Composite Metamaterials: Design, Fabrication, and Characterization

**DOI:** 10.3390/ma13214749

**Published:** 2020-10-23

**Authors:** Alexa Rizzo, Claudia Luhrs, Brian Earp, Dragoslav Grbovic

**Affiliations:** 1 Mechanical and Aerospace Engineering Department, Naval Postgraduate School, Monterey, CA 93943, USA; akrizzo@gmail.com (A.R.); ccluhrs@nps.edu (C.L.); earp@usna.edu (B.E.); 2 Physics Department, Naval Postgraduate School, Monterey, CA 93943, USA

**Keywords:** metamaterial, carbon nanotube, electromagnetic interference shielding, microwave absorption, conductive epoxy composites

## Abstract

In this study, carbon nanotube (CNT) epoxy composite films were fabricated, characterized, and tested as resonant, plasmonic metamaterials. CNT–epoxy formulations, containing diverse CNT loadings, were fabricated and templates were used to generate repeating arrays of squares of diverse dimensions. Their absorption characteristics were characterized by collecting free space reflectivity data in the microwave band, using an arch setup in an anechoic chamber. Data were collected from 2 to 20 GHz. The materials behavior was modeled using a standard unit-cell-based finite element model, and the experimental and calculated data were compared. The experimental results were successfully reproduced with appropriate adjustments to relative permittivity of the composite films. This research demonstrates the ability to use CNT-based conductive composites for manufacturing metamaterials, offering a potentially lighter-weight alternative in place of traditional metal films. Lower conductivity than other conductors causes a widening of the absorption curves, providing a wider band of frequency absorption.

## 1. Introduction

### 1.1. Electromagnetic Interference (EMI) Shielding

With advances in digital communications, EMI shielding has become vital for maintaining proper functionality of electronic systems [1,2,3,4]. EMI shielding uses conductive materials to prevent electromagnetic (EM) waves from propagating into undesirable locations. The bulk of undesirable EM radiation can be attributed to developments in telecommunication technology [5]. Effective EMI shielding must reduce the harmful radiation penetrating the system by reflecting or absorbing it [1,2]. When an EM wave is incident on a material, the non-reflected and non-transmitted portion of the wave is dissipated in the form of heat [5]. The magnitude of energy dissipation at a given frequency is directly correlated to shielding effectiveness (SE) and will depend greatly on the material’s electric permeability (µ’) and permittivity (ε’) [2,6,7].

### 1.2. Metamaterials as EM Absorbers

The commonly accepted definition of a metamaterial is an artificially structured material, designed, with some exceptions, in a repeatable structural pattern, which can be designed to manipulate different physical phenomena, such as light or sound [8,9]. The structures are designed for specific shapes and dimensions to affect the EM wave response at a targeted wavelength/frequency. This approach has shown a lot of promise in offsetting the negative effects of unwanted microwave radiation [10,11,12].

**Figure 1:**
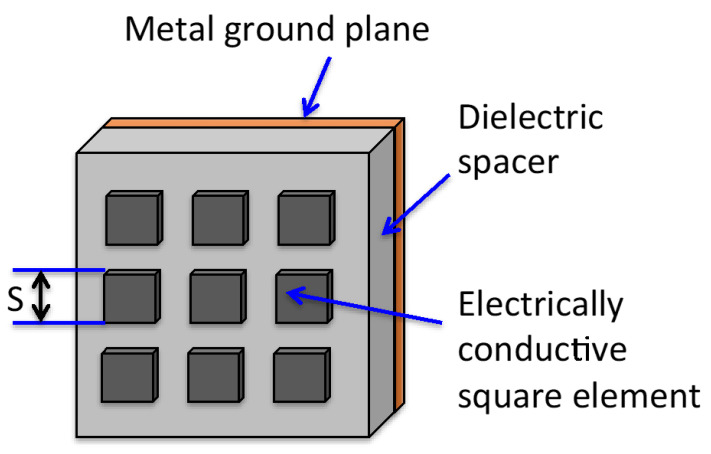
Schematic of a conductive square array metamaterial.

Traditional materials exhibit microstructures and properties based on their individual atomic and molecular makeup. When exposed to EM radiation, the material’s electrons, and possibly atomic lattice sites, experience a force and increase in kinetic energy [9]. The increased energy is exhibited in the form of increased temperature, as some of absorbed energy turns into heat. In a metamaterial, the EM properties of the sample depend less on the material composition, and more on the structured design and geometry of the metamaterial sample [10,13,14,15]. The metamaterial patterns must be smaller than the wavelength of incident EM radiation. A common metamaterial configuration that has been studied extensively is the conductive square array [10,15,16,17,18]. Figure 1 provides a schematic of this configuration, highlighting the periodic patterned conductive material layer, the dielectric spacer, and the ground plane. In this configuration, the array of squares of size *s* is patterned in a conductive layer. This layer is separated from a continuous conductive layer, called the ground plane, by a dielectric layer.

A resonant reaction between the dielectric and the conductive material occurs once the metamaterial is exposed to EM radiation of a particular frequency, specific to the structural dimensions. The free electrons within the conductive layer become excited and eventually match the frequency of the incident EM wave. The matching frequency responses of the electrons and radiation generate surface plasmons that decay into the dielectric and conductive materials [9]. Resistive losses in both the metal and dielectric materials convert the incident radiation energy into joule heating, resulting in radiation absorption [9].

Popular materials used for EMI shielding include carbon steel, tin plated steel, copper, Cu-Ni-Ag alloys, and aluminum [19]. Most of these materials come with disadvantages such as high weight, susceptibility to corrosion, high stiffness, high cost, and low tuning capabilities for shielding effectiveness [2,4,20]. Several studies have looked into use of carbon nanotube (CNT)-based nanocomposites for shielding applications [2,20,21,22,23,24,25,26]. CNT-reinforced polymer composites have also been studied as radar-absorbing materials in the microwave range [6], as the composite is capable of absorbing incident microwave radiation and attenuating it via the internal CNT electrons. Due to absorption being a function of material conductivity, permittivity, permeability, and thickness, incorporating conductive fillers into polymeric matrices can lead to better-absorbing materials [3,4,5,6].

### 1.3. CNT Composites in EMI Uses and Microwave Absorption Studies

Electrically conductive composites, specifically produced using insulating polymeric materials and additive conductive fillers, have been in high demand, due to their exceptional properties and potential for various applications, including but not limited to: EMI shielding, electrostatic dissipation (ESD), sensor components, and bio-medical devices [1,21,27,28,29]. Depending on the end-use applications for the composite material, a varying resistivity value may be required, with ESD and EMI usually being in the semiconductor range [30,31].

Microwave absorption studies in CNT composites include those of Saini et al. [4], who used polyanaline (PANI), popular due to its environmental stability and low costs, in place of an insulating polymer. Most of the reported CNT/PANI composite experiments used higher CNT loadings (5%, 10%, 15%, 20%, and 25% CNT by weight). Their microwave absorption characterization employed 2 mm thick composite rectangular pellet samples, placed on a metal backing, and performed measurements between 2.4 GHz to 18 GHz using a vector network analyzer (VNA). Using classical transmission line theory and the scattering parameters detected from the VNA, the samples were characterized in terms of absorbance, reflectance, and transmittance. The overall results showed that CNT/PANI composites proved useful for microwave absorption purposes over the Ku-Band in the microwave frequency range (12–18 GHz), with shielding effectiveness up to 28% [4].

Wang et al. [7] performed a similar study but using epoxy resins as the matrix material and lower CNT loading in the composites (1–10% by weight). Their work was focused on microwave absorption responses over the 2 to 20 GHz frequency range. In order to characterize the microwave absorption properties of the samples, the study by Wang et al. used a VNA in conjunction with classical transmission line theory; absorbance values were calculated along real and imaginary parts of the permittivity, loss tangent, and complex permeability of the CNT/epoxy composite samples. Their overall results showed that absorption efficiency of the CNT/epoxy composites strongly depended upon frequency of incident radiation and CNT loading in the epoxy resin. Higher loadings of CNTs outperformed the lower loadings, up to 8%, which was deemed the optimal loading in this experiment, as it achieved a microwave absorption ratio up to 26% at 20 GHz. They also determined that the bulk of the radiation absorption over the entire 2 GHz to 20 GHz frequency range is due to the dielectric loss of the composite samples. Since CNTs and epoxy resin have poor magnetic properties, lower values of both the real and imaginary parts of permeability were measured and contributed to the sample dielectric losses [7].

Che et al. [32] performed a microwave absorption study on CNT/epoxy composites for loadings lower than in [4,7], ranging from 0.125% to 2.0% using three different commercially available CNTs. Two different composite fabrication protocols were performed; one aided by surfactants and another using ball-milling to correlate dispersions techniques to electrical conductivity and microwave absorption characteristics. Composite samples used for microwave absorption measurements were fabricated into 2–3 mm thick single layer square sheets and studied backed with a metal plate. Using a VNA, the scattering parameters were measured, and absorption was calculated in similar way as in previous studies. The results were reported in terms of reflection loss, equivalent to shielding effectiveness due to reflection. The study showed that loadings as low as 0.125%, microwave absorption can be achieved, using the two fabrication methods and dispersion techniques mentioned. Absorption efficiencies up to 97% were reported.

Percolation theory suggests that if conductive fillers are introduced into a non-conductive matrix above the percolation threshold, these fillers will form a continuous conductive path throughout the composite [1,2,33,34,35]. At filler loadings below 1% by weight, electrical conductivity values tend to be achieved through percolation networks that form when fillers of large aspect ratios are employed or by the existence of zones devoid of CNTs, while still maintaining a conductive path. As an example of the later, Earp et al. studied CNT epoxy composites with loadings below 1% CNT, establishing that loadings as low as 0.1% present adequate conductivities for EMI applications when excluded volumes are formed within the composite matrix. It was found that above 0.10% CNT loading, a continuous percolation network is formed, with the concurrent dramatic increase in conductivity [33]. Subsequent work showed that below 0.10% the mechanism of conduction is dominated by a capacitive-like behavior [36]. Similar findings were published by others providing a proof of concept that CNT conductive composites, even at very low CNT loadings, have potential for EMI applications [32,34,37,38,39].

The present work aimed to use epoxy composites with very low loadings of CNTs (0%, 0.014%, 0.10%, 0.20%, and 0.75%), as the conductive element, arranged in a repeating pattern that will, along with a dielectric sheet, constitute a metamaterial. The objective of the work herein was to provide a proof of concept that CNT/epoxy composites could also be employed as metamaterial constituents and not only for continuous networks. We analyzed several tailored square arrangements of some of those composites. Thus, the next sections present information about how diverse CNT epoxy composite array dimensions were generated and tested and how the experimental data compare with finite element models.

## 2. Materials and Methods

### 2.1. Fabrication

Composites were fabricated with multiwall carbon nanotubes (MWNCT) provided by Nanocomp Technologies, Inc (Parent organization: Huntsman Corporation, Merrimack, NH, USA) and a commercial-off-the-shelf, space-rated epoxy resin (Henkel Loctite Hysol EA9396 Aero, Henkel Corporation, Dusseldorf, Germany). The MWCNTs from Nanocomp were produced using a chemical vapor deposition process with an iron catalyst to generate large CNT sheets. The iron catalyst was not removed from the CNTs. The CNT sheets were then ground into interwoven CNT bundles of approximately 0.05 mm diameter and 1.0 mm length using a high-speed attritor and industrial burr mill [40]. The epoxy resin is a two-part system consisting of a Part A epoxy and Part B hardener that are combined in a 100:30 ratio, respectively. CNTs were weighed and added to the Part A epoxy in order to achieve the desired CNT loading weight percentages. Given that the CNT and resin do not present compatibility or stability issues, there was no need to activate them or perform prior treatments before mixing. Part B epoxy was then added, and the sample was mixed with a dual asymmetrical mixing process using a FlackTek DAC 150.1 FVZ-K speed mixer (FlackTek, Landrum, SC, USA). Following two low-speed mixing cycles at 1200 rpm and 2500 rpm, the sample was allowed to cool, to prevent heat buildup, and a vacuum was applied using a Buehler LTD 20-2850-160 vacuum pump (Buehler LTD, Lake Bluff, IL, USA), to minimize porosity following curing. Three high-speed mixes at or above 3000 rpm were then conducted, with a cooling period and vacuum application after each mix. No additional heat was applied during the mixing process. Each mixing cycle lasted one minute.

**Figure 2:**
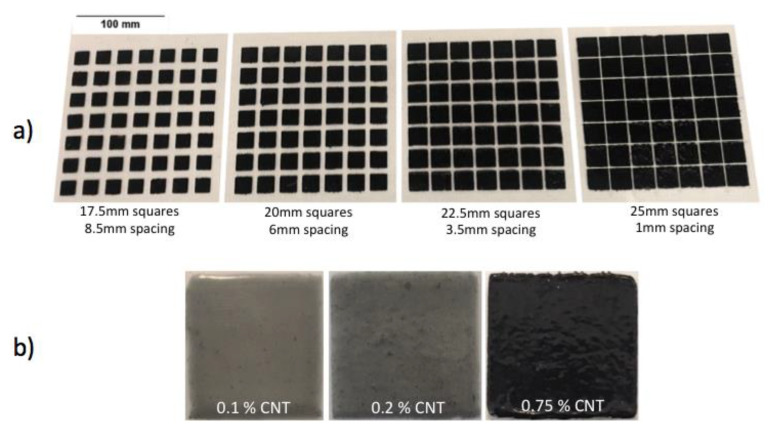
(**a**) Carbon nanotube (CNT)/EA9396 0.75% samples fabricated with diverse dimensions and (**b**) visual comparison of CNT/EA9396 single square composites with 0.1, 0.2, and 0.75% CNT loading.

For every loading value of CNT/epoxy composite (0%, 0.014%, 0.10%, 0.20%, and 0.75%), a sample was created in four different-sized metamaterials based off a 26 mm pitch unit cell (17.5 mm squares/8.5 mm spacing, 20 mm squares/6 mm spacing, 22.5 mm squares/3.5 mm spacing, 25 mm squares/1 mm spacing). The 26 mm pitch was selected to be adequate for the frequency range under study and was kept constant for all samples to allow stacking of individual sheets to produce multi-band metamaterial [10]. The produced CNT/epoxy composite was applied over an insulating substrate using a 200 μm thick laser-cut acetate sheet as a template to create the required unit cell spacing. The samples were then furnace-cured at 66 degrees Celsius for one hour, per EA9396 manufacturers guidelines (reference—EA9396). Some of the metamaterials produced on top of acrylic substrates are shown in Figure 2. It is worth noting that the viscosity of the samples, as well as their optical transparency, varied with CNT loading.

### 2.2. Microstructural and Electrical Characterization

Microstructural characterization of the CNTs used in the generation of the CNT/epoxy composites was performed employing a Zeiss Neon 40 field emission SEM (Carl Zeiss Inc., Thornwood, NY, USA). Samples were analyzed with an accelerating voltage up to 20 kV and an aperture size of 30 μm over a range of magnifications between 100× and 55,000× that supported observation of CNT bundle shapes and sizes.

In order to determine electrical properties of the generated CNT/epoxy composites, material from the same batch used to create the metamaterials, seen in Figure 2, was applied to a four-point circuit board. Specifically, a thin layer strip of composite material was applied to the circuit board and then cured in the same manner as the metamaterials [33]. The four-point circuit board was then analyzed using a 2400 Keithley Source Meter (Tektronix, Inc., Beaverton, OR, USA) as the current source and a digital multi-meter to measure the voltage drop across each sample for various applied currents. Using sample thickness, the resistivity of each sample was calculated.

In addition to the method described above, following the curing of the CNT/epoxy composite on the acrylic substrate, a rough two-point probe method was used to measure surface resistance and verify that the material remained conductive when applied in the desired patterns for optical characterization.

### 2.3. Optical Characterization

**Figure 3:**
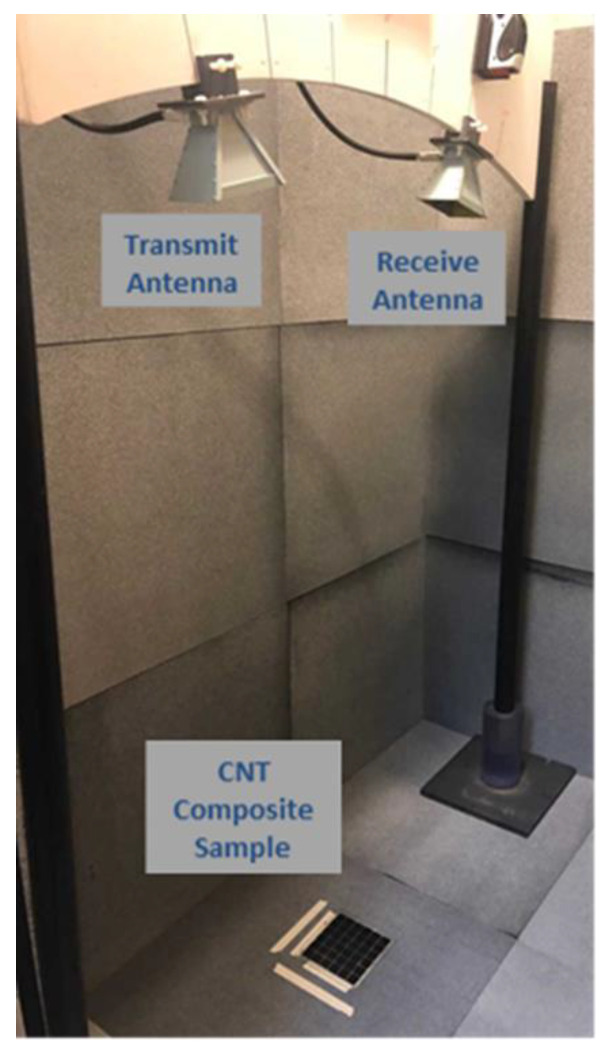
Arch setup used to measure microwave spectral response.

Absorption characteristics for the composite samples were conducted by collecting free space reflectivity data in the microwave band, using a Naval Research Laboratories (NRL)-type arch setup in an anechoic chamber [4,41], with walls completely covered by layers of lightweight, flexible, microwave-absorbing foam, shown in Figure 3. The NRL arch consists of two Cobham H-1498 horn antennas that are equally distanced from the center of the arch and positioned at symmetric angles (10°) that are approximately two meters above a flat reflecting surface. This surface served the purpose of calibrating the “perfect reflector” as well as the ground plane, making the configuration similar to that in the studies described in [4,32] that used backing for the material. An Anritsu Shockline MS46122b-020 2-port vector network analyzer, covering frequency ranges from 1 MHz to 20 GHz, was used. The test setup included transmitting a signal directly to the composite sample via one horn antenna and collecting the reflected signal with the second antenna. This device can collect measurements in terms of scattering parameters in the frequency domain or the time domain. The signal collected is a ratio of power in (receiving antenna) to power out (from transmitting antenna). For this research, all data were collected in terms of this ratio, from 2 GHz to 20 GHz.

The reflectivity data were gathered for each sample, placed on a metal backing, and allowed calculation of the absorptivity of the composite sample using the relationships of classical transmission line theory in a similar way than the studies previously mentioned in [4,7,32]. The reflectivity was determined by measuring the transmitted power between the transmitter and receiver antenna of the vector network analyzer and calculated as T_s_/T_background_. The variable T_s_ is the reflected signal transmitted from the transmitter antenna, reflecting from the sample, placed on a metal baking of the same footprint (serving as ground plane in Figure 1), and received by the receiver antenna, while the variable T_background_ is the signal transmitted from the transmitter antenna, reflecting from the metal backing (serving as near-perfect mirror) and received by the receiver antenna as in [10]. The metal backing in this experimental setup was a piece of flat copper that was cut to the exact size and placed in the position of the composite sample substrates (203 mm × 254 mm). The T_background_ was accounted for in the experimental data via initial calibration of the equipment. The transmittance (T) of the sample/metal backing system was assumed to be zero, as the metal backing in the metamaterials was thicker than the skin depth for all frequencies studied.

## 3. Finite Element Modeling Parameters

Previously developed models for resonant metamaterials at NPS, for THz and microwave range, and described in detail in [10,16,18] were used to analyze the experimental measurements of absorption. Finite element (FE) models were created using the radio frequency module of COMSOL Multiphysics software (COMSOL, Burlington, MA, USA). Due to the periodic nature of the metamaterial structures, simulation of a unit cell, using periodic (Floquet) boundary conditions, on the sides and ports on top and bottom of the unit cell, can provide sufficient analysis for the larger metamaterial.

Once the model is solved, sweeping the frequencies to match the experimental setup, the absorption can be retrieved either as 1-R or alternately, by integrating all of the Joule heating losses in the metamaterial. The material properties used in the model are displayed in Table 1. Other than the material properties, dimensions, and frequency range, no other aspects of the model were modified. Parameters that were explicitly measured, such as the conductivity of the composite (described in Section 2.2) and material thicknesses and square dimensions were maintained as fixed.

Initial values for acrylic refractive index (1.65) and extinction coefficient (1.4) were obtained from [42,43] and adjusted, through iterative process, until the experimental absorption pattern of plain acrylic matched that obtained in the simplified model. The fixed values are shown in Table 1.

## 4. Results and Discussion

### 4.1. CNT Epoxy Composites Microstructural Characterization

**Figure 4:**
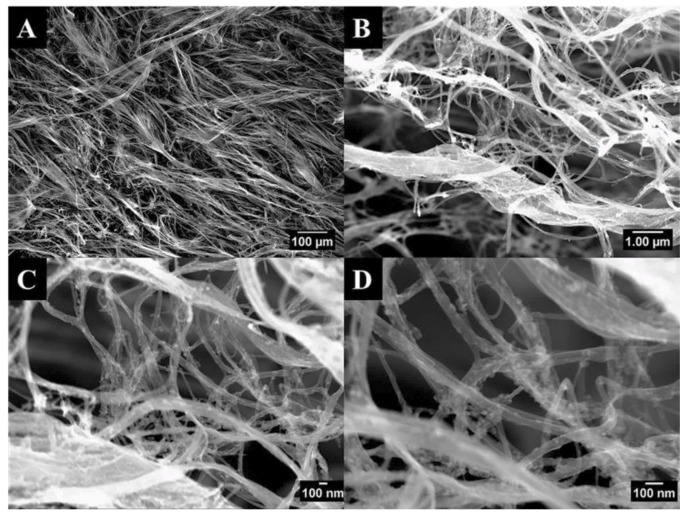
SEM images of the as received CNTs at varying magnifications (**A**) 100×, (**B**) 10000×, (**C**) 25000×, and (**D**) 55000×.

Figure 4A–D shows the as-received CNT bundles from Nanocomp. The bundles are made up of intertwined individual CNTs, as seen in Figure 4, and have typical widths of up to 100 μm and lengths on the order of millimeters. At the highest magnification in Figure 4D, some individual CNTs can be observed as well as small Fe particulates that served as a catalyst during the CNT fabrication process. Individual CNTs had an average diameter of 30 nm. The CNT distribution within the polymeric matrix was previously studied and described in detail [33]. As mentioned in the latter, the location of the CNTs within the matrix is quite different for each loading studied; samples loaded with 0.75 wt.% CNTs show a network of interconnected CNTs, which expands in all directions and alternates with CNT-free zones. In contrast, the strands of nanotubes in samples loaded with 0.014 wt.% CNTs expanded in different directions and seemed isolated from each other by distances that extended from a few to tens of μm.

### 4.2. Optical Characterization of CNT Epoxy Metamaterials

**Figure 5:**
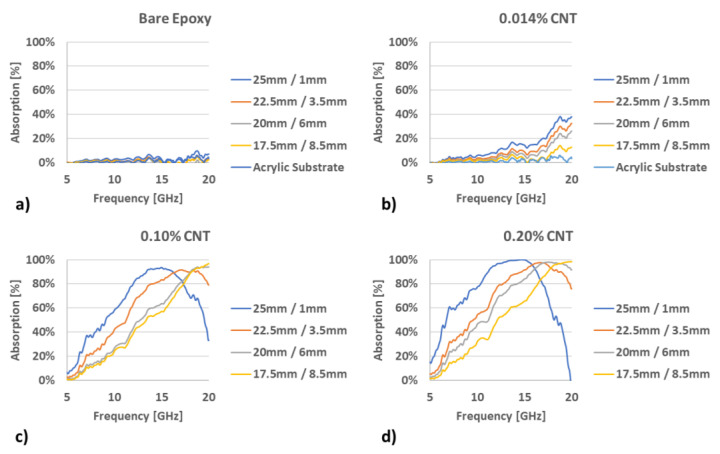
Percent absorption of CNT/epoxy composites at varying loadings: (**a**) 0%, (**b**) 0.014%, (**c**) 0.10%, and (**d**) 0.20%, respectively.

A summary of results from the arch setup can be seen below in Figure 5, which provides the absorption spectra for the various CNT loadings that were part of the research. While composites were generated with five different CNT loadings (0%, 0.014%, 0.10%, 0.20%, and 0.75%), only four were evaluated after production. The 0.75% sample was excluded due to high sample viscosity, which resulted in a significantly higher degree of roughness after application to the acrylic plate. This in turn led to a large degree of scattering, resulting in unusable absorption plots.

The results were consistent in trend to the results of [7,32]. They show that the bare epoxy absorption response remains negligible; but as the conductive CNT filler is inserted into the epoxy composite increased from 0.014% to 0.10%, the absorption response drastically increases. The sample with 0.014% CNT presents a very small percentage of absorption, consistent with previous work that shows an incomplete conductive network at such extremely low loadings, thus, low conductivity. Remarkably, those specimens still exhibit an absorption above 20% at frequencies of 17 GHz or higher. In addition, the data presented for 0.10% and 0.20% CNT loadings shows a clear dependence of absorption characteristics on the CNT loading (which correlates to material conductivity) and the metamaterial geometries, with peak responses (up to 97%) exhibited in the microwave region of 2 to 20 GHz for the largest square sizes (25 mm squares/1 mm spacing). Composites with higher loadings and higher conductivities exhibited a shift in the peaks to lower frequencies. In addition, observed peaks were broader than what is typically seen when regular metals are used for the patterned layer.

When the absorption properties of the metamaterials presented in this work are compared to the composites studied in [4,7,32], the composites generated using Nanocomp´s CNT bundles outperform those significantly (note that some of them are not optimized for absorption but achieve their shielding effectiveness from reflection). Whereas the CNT/EA9396 metamaterials achieved close to 100% absorption at 0.10% and 0.20% loading, those composites’ absorption ranged from 6.3% wideband absorption at 25% loading in [4] and 26% at 8% loading in [7] to 98% at 0.25% and 2–3 mm thickness but at a much narrower band in [32]. Table 2 shows a comparison of the best-performing materials in those studies.

From Table 2, it can be seen that for absorption purposes at a wider band, the metamaterials studied herein showed a much wider absorption band than composites in other reports. While Nanocyl NC7000 tubes (Nanocyl SA, Sambreville, Belgium) did achieve near-perfect absorption, the band of absorption was much narrower than that of metamaterials presented in this work. Additionally, the low percentage of CNT used in this study has the potential to drive down the overall material cost, since the amount of CNTs used is less.

The distribution of the CNT bundles within the epoxy matrix, which form conductive networks in between empty spaces of variable dimensions, might, to a certain extent, be responsible for the shape of the absorption peaks. Previous work by our team determined, using electron microscopy, that the composite microstructure, despite forming an interconnected conductive path, contains zones of approximately 9–13 μm devoid of CNTs [33]. The distribution of those, across the sample varies in the three dimensions, thus, might constitute a “defect”, which promotes a different optical response.

### 4.3. Finite Element Model Results

**Figure 6:**
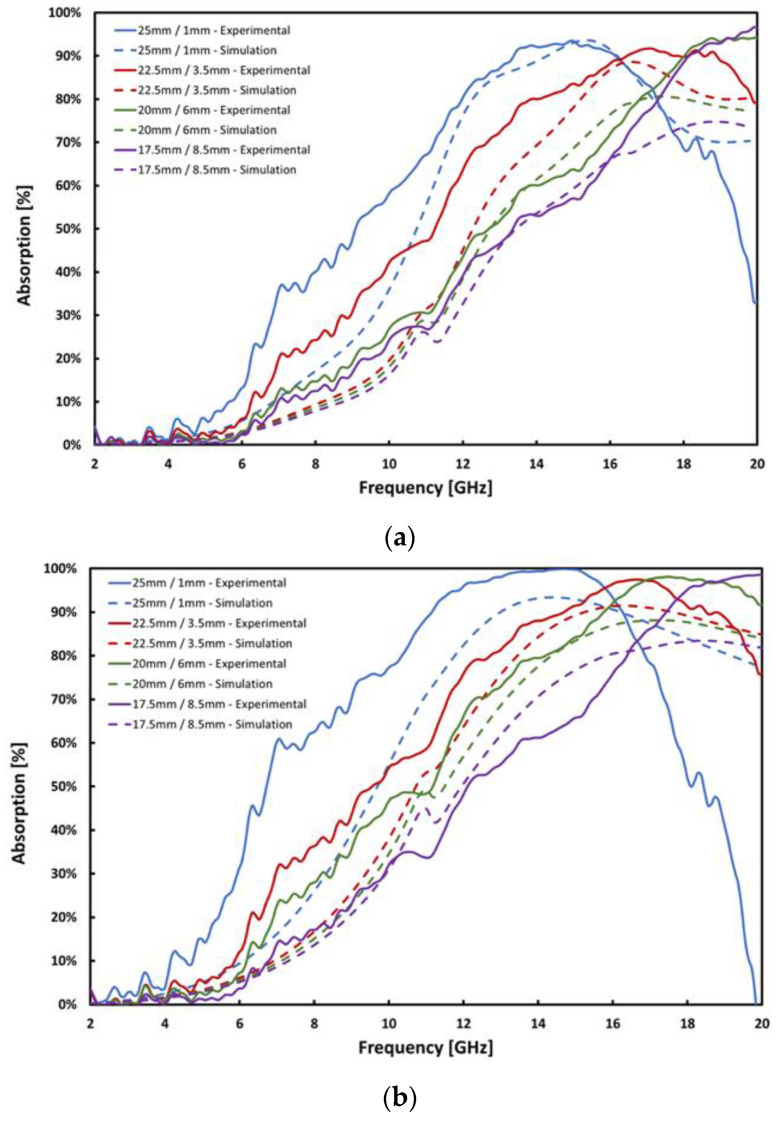
Comparison of model and experimental results of (**a**) 0.10% CNT/EA9396 and (**b**) 0.20% CNT/EA9396.

Results were analyzed using a COMSOL Multiphysics finite element model of metamaterials developed at NPS [10,16,18]. All of the parameters able to be experimentally measured, such as resistivity, thicknesses, and square dimensions, were fixed in the model. These are shown in Table 1. In order to achieve agreement between experimental measurement and finite element model, relative electrical permittivity, ε_r_, was used as a fitting parameter. There are articles in the literature that report permittivity of 1 < ε_r_ < 44 for conductive composites [28,30], and those values were used as starting points in analysis. Using ε_r_ of 35 for 0.10% loading and ε_r_ of 30 for 0.20% loading provided acceptable agreement between experimental measurements and the model. The results are shown in Figure 6.

The broadening of the resonant absorption peaks is consistent with the lower conductivity of the patterned layer. Broadening of the absorption band is a positive benefit, as it increases the range of frequencies that the material can absorb. Lower conductivity (0.862 S/m) than metal conductors may have contributed to the widening of the absorption curves, providing a greater band of frequency absorption. Larger square arrangements showed absorption bands at lower frequencies, as seen in previous work [15,18]. In addition, as observed experimentally in THz band [18], the model shows a decrease in absorption magnitude as the square dimensions (and resonant frequency) decrease, everything else being constant. This is related to the decreasing fill factor. However, experimental data deviate from that. One possibility to consider exploring in future work is that the dielectric constant is frequency-dependent and that the change in it compensates for the drop in fill factor.

## 5. Conclusions

This research demonstrates the ability to use CNT-based electrically conductive composites at loadings below 1% for building metamaterials with absorptions within the 2–20 GHz range, which offers a potential lighter weight and lower cost alternative, in place of traditional metal films. The experimental results were successfully reproduced using a standard unit-cell-based finite element model with appropriate adjustments to conductivity and relative permittivity of the composite films.

CNT epoxy composites seem to be promising candidate metamaterials where conductivity of the conductive portion of a metamaterial can serve as a tuning parameter for the optimization of absorption bands in addition to material geometry.

Previously developed models for traditional resonant metamaterials seem to be adequate to model the behavior of CNT epoxy composite metamaterials, provided the material parameters, such as composite conductivity and >1 dielectric constant, are adjusted. A more in-depth study of frequency dependence of dielectric constant would be valuable.

## Figures and Tables

**Table 1:** Material properties used in the finite element model for the two composites.

	0.10% CNT/EA9396	0.20% CNT/EA9396
Composite conductivity [S/m]	0.461	0.862
Composite relative permittivity	35	30
Composite thickness [mm]	0.2	
Acrylic board thickness [mm]	1.5	
Acrylic refractive index	1.7	
Acrylic extinction coefficient	1	

**Table 2:** Comparison of selected composite materials performance.

Material	CNT Weight (%)	Absorption [%]	Total Thickness [mm]	Conductivity [S/m]
CNT/PANI composite [4]	25	6.3	1.5	2 × 10^3^
MWCNT/Aero Marine 300/21 [7]	8	26	2	Not reported
Nanocyl NC7000 CNT/epoxy [32]	0.25	99	2–3	3.87 × 10^7^
Resonant metamaterial (Current work) (0.2 wt.%)	0.2	100	1.7	0.862
